# Novel Methods to Enhance Precision and Reliability in Muscle Synergy Identification during Walking

**DOI:** 10.3389/fnhum.2016.00455

**Published:** 2016-09-15

**Authors:** Yushin Kim, Thomas C. Bulea, Diane L. Damiano

**Affiliations:** Functional and Applied Biomechanics Section, Rehabilitation Medicine Department, National Institutes of Health, BethesdaMD, USA

**Keywords:** muscle synergy, motor module, walking, motor complexity, coordination

## Abstract

Muscle synergies are hypothesized to reflect modular control of muscle groups via descending commands sent through multiple neural pathways. Recently, the number of synergies has been reported as a functionally relevant indicator of motor control complexity in individuals with neurological movement disorders. Yet the number of synergies extracted during a given activity, e.g., gait, varies within and across studies, even for unimpaired individuals. With no standardized methods for precise determination, this variability remains unexplained making comparisons across studies and cohorts difficult. Here, we utilize *k*-means clustering and intra-class and between-level correlation coefficients to precisely discriminate reliable from unreliable synergies. Electromyography (EMG) was recorded bilaterally from eight leg muscles during treadmill walking at self-selected speed. Muscle synergies were extracted from 20 consecutive gait cycles using non-negative matrix factorization. We demonstrate that the number of synergies is highly dependent on the threshold when using the variance accounted for by reconstructed EMG. Beyond use of threshold, our method utilized a quantitative metric to reliably identify four or five synergies underpinning walking in unimpaired adults and revealed synergies having poor reproducibility that should not be considered as true synergies. We show that robust and unreliable synergies emerge similarly, emphasizing the need for careful analysis in those with pathology.

## Introduction

Muscle synergies are purported to represent functional neural commands descending from groups of motor neurons in the motor cortex and spinal cord (Machado et al., 2015; Overduin et al., 2015). If this is the case, one would anticipate synergy number and structure to be consistent for repetitive tasks performed in the same biomechanical context (i.e., consecutive gait cycles) in healthy adults (Ivanenko et al., 2006; Safavynia and Ting, 2012; Ting et al., 2015). Some degree of consistency in synergy number has been reported in normal gait despite differences in body weight support or gait speed within subjects and number of electromyography (EMG) channels between subjects (Ivanenko et al., 2004). Based on this consistency, common synergy structures between individuals have been found using clustering analyses (Steele et al., 2015). Precise identification of synergies is particularly important when analyzing gait pathology. Synergy number is reportedly reduced by one or more levels in many individuals with brain injury, an observation attributed mainly to merging of synergies (Allen et al., 2013). Reduction in synergy number has also been correlated with clinical measures indicating diminished function (Bowden et al., 2010). This leads to the conclusion that the number of muscle synergies and their structure are meaningful features in clinical populations which may be indicative of motor control capacity and its reduction in pathology (Ting et al., 2015).

However, previous studies have shown variability in the number of synergies extracted during walking in healthy individuals, ranging from as few as 2 or 3 to as many as 6 (Clark et al., 2010; Rodriguez et al., 2013). When applying the same analysis procedure as in healthy individuals, those from clinical populations have shown a range of 2 to 5 synergies (Clark et al., 2010; Rodriguez et al., 2013). While lower synergy number has been related to lower functional assessment scores (Bowden et al., 2010), this overlap demonstrates the variability of functional capability at a given level. Thus, accurate selection of synergy number is critical for studying synergies in the context of clinical assessment of function.

Currently no standardized criteria or advanced methods have been established for computing the precise number of highly reliable synergies from EMG recordings. One of the most common methods is application of a cut-off threshold based the variance accounted for (VAF). VAF is computed by comparing EMG reconstructed from one or more synergies to the original EMG. The minimal number of synergies, also termed the minimal level, is selected using a threshold criterion for VAF, which has ranged from 80 to 95% in previous studies (Dominici et al., 2011; Gizzi et al., 2011; Rodriguez et al., 2013). Discordance over the VAF threshold value intensifies the confusion over which level is correct. Moreover, it has not been justified that a single cutoff value should be applied across all individuals. In addition to VAF threshold, other methods have been applied to determine the minimal synergy level, including likelihood ratio tests and Bartlett, Akaike, Bayesian, and Laplacian information criteria, although these measures may overestimate the number of synergies, especially in the presence of signal noise (Tresch et al., 2006). Another study identified minimal level by linear fit of R^2^ as a function of synergy number (Cheung et al., 2005; Tresch et al., 2006), yet this method still relies on setting a threshold for mean squared error between the *R*^2^ curve and the linear fit. Other studies have used bootstrapping techniques to resample EMG data sets multiple times with replacement and recalculate VAF (Sawers et al., 2015) or *R*^2^ value (Cheung et al., 2009); the minimum synergy number is identified as the value at which the 95% confidence interval exceeds 90% VAF or *R*^2^. By incorporating resampling of individual strides, the synergy number identified by these bootstrapping approaches can control for stride-to-stride variability in the synergy number whereby elevated synergy variability would result in decreased VAF (or *R*^2^) values at the 95% confidence bounds, and thus an increased synergy number. These approaches, however, do not quantify the stride-to-stride reliability of individual muscle synergies.

The number of synergies can also be influenced by other steps in EMG collection and data processing such as low-pass filtering techniques (Hug et al., 2012), the number and choice of muscles collected (Steele et al., 2013), and even how EMG data sets are preprocessed before synergy extraction, e.g., averaging or concatenating (Oliveira et al., 2014). The focus of this study, however, is on developing criteria that can be applied subsequent to VAF threshold technique to validate the choice of minimal synergy level, which is a critical step in studies utilizing muscle synergy analysis to characterize neural control of a movement.

Here, we introduce a new method to extract synergies during walking. We hypothesized that fundamental synergies underlying walking function would have high stride-to-stride reliability across consecutive gait cycles because essential motor activities are repeated with low variability in purposeful behaviors (Cullins et al., 2015). Moreover, by incorporating *k*-means clustering with an intra-class correlation analysis, our method provides a quantitative way to identify reliable synergies beyond the minimal level required to describe walking, which may have been rejected as noise in previous studies (Cheung et al., 2005; Chvatal and Ting, 2012).

## Materials and Methods

### Participants

Nine healthy volunteers (8 right side dominant and 1 ambidextrous; 4 females and 5 males; mean age of 26.1 ± 7.1 years, mean body mass and height of 78.4 ± 12.3 kg and 173.2 ± 10.5 cm, respectively) participated in the experiment. Exclusion criteria included a history of musculoskeletal surgery within 6 months, unresolved musculoskeletal injury, or neurological dysfunction. All subjects provided written informed consent before participation in the Institutional Review Board approved protocol.

### Procedure

Participants were allowed to familiarize themselves to walking on the treadmill (Bertec TM-06-B, Columbus, OH, USA) by walking for 2 min at their self-selected comfortable speed while looking straight ahead. After familiarization with the treadmill and pace, subjects walked at their preferred speed for 5 min while EMG and kinematic data were recorded.

Electromyography signals were recorded with surface electrodes using a wireless EMG system (Trigno Wireless, Delsys, Boston, MA, USA). Before electrode placement, the skin was abraded and cleaned with alcohol. The electrodes were attached by tape and then secured more closely to the skin surface by non-adhesive wrap (Coflex, Andover Healthcare, Inc., Salisbury, MA, USA) to reduce motion artifact. EMG signals were sampled at 1000 Hz from the following muscles on the both legs: tibialis anterior, medial gastrocnemius, rectus femoris, and medial hamstrings. Kinematic data were collected using 10 motion capture cameras and synchronized with EMG data (Vicon, Lake Forest, CA, USA). Reflective markers were placed on the skin over specific anatomic locations on the pelvis and lower extremities to measure angular displacement of hip, knee, and ankle joints. Motion capture data were processed oﬄine with Visual 3D software (C-Motion, Germantown, MD, USA) to identify gait events (heel strike and toe-off). Individual gait cycles were identified using right heel strike events.

### Data Analysis

**FIGURE 1:**
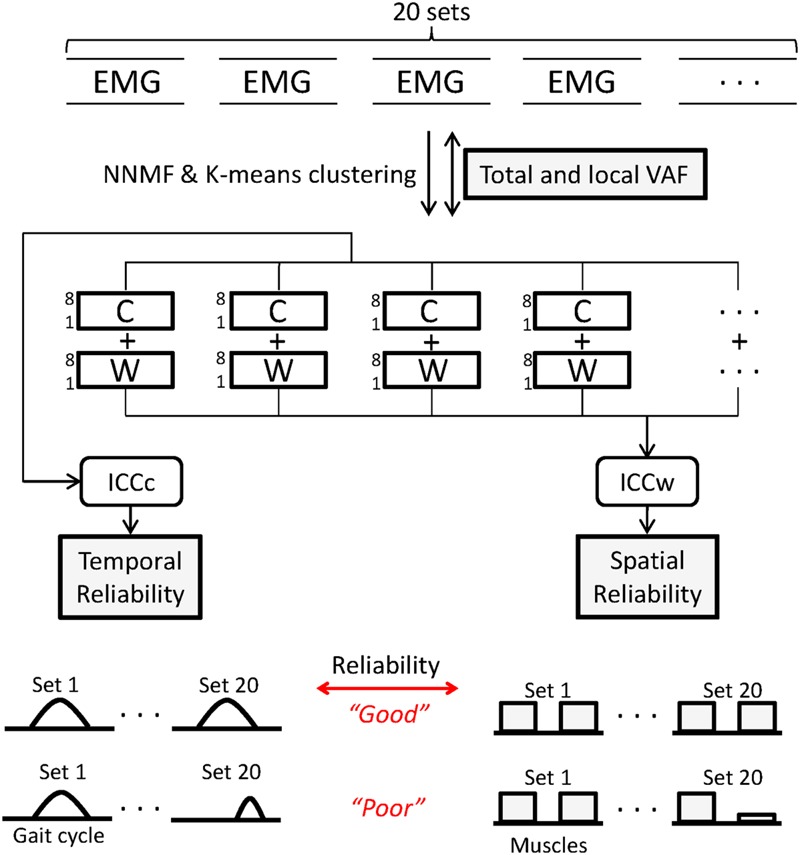
**Twenty sets of electromyography (EMG) matrices comprising 20 consecutive gait cycles were used for analysis.** Non-negative matrix factorization (NNMF) was used to extract muscle synergies from levels 1 to 8. Agreement of EMG reconstructed from C and W matrices and original EMG was calculated using variance accounted for (VAF) for all muscles (total) and for individual (local) muscles. Within each level, *k*-means clustering was used to identify similar synergies across the 20 sets. Intraclass correlation coefficients of W (ICCw) and C (ICCc) for the 20 sets was computed to quantify reliability of muscle synergies. For each parameter, a schematic representing good and poor states of the indices is shown.

The mean number of strides for the 5 min walking trial across subjects was 270 ± 7.8. For each participant a block of 20 consecutive gait cycles was selected for analysis starting with the 100^th^ stride. Each surface EMG channel was demeaned, high-pass filtered (2nd-order, 30 Hz), full-wave rectified, and low-pass filtered (2nd-order, 5 Hz) to create the linear envelope; the low-pass cutoff frequency was based on the approximate walking cadence of 1 Hz, as recommended in Hug (2011). Next, each EMG envelope was segmented by gait cycle using synchronized kinematic data. EMG data for each gait cycle were resampled to 100 points (1-100% from right heel strike to right heel strike) using cubic spline interpolation. Muscle synergies were computed using non-negative matrix factorization (NNMF) as described previously (Lee and Seung, 1999). Prior to NNMF, the 20 sets of EMG matrices were normalized by individual peak activation, so that all EMG data had amplitude in the range of 0 to 1. After normalization, NNMF was applied 20 times to each of the 20 EMG matrices (once per gait cycle) as shown in **Figure 1**. We chose to extract synergies from individual gait cycles because this process was shown to result in higher reconstruction quality than muscle synergies from averaged or concatenated EMG (Oliveira et al., 2014) and variation in muscle activation across gait cycles could be lost in averaging process (Steele et al., 2015). The 20 sets of muscle synergies were each computed according to the following formula:

(1) $${\text{EMG}}_{0}=\sum_{\text{i}=1}^{\text{n}}W_{\text{i}}C_{\text{i}}+e,{\text{EMG}}_{\text{r}}=\sum_{\text{i}=1}^{\text{n}}W_{\text{i}}C_{\text{i}}$$

where EMG_o_ is the recorded muscle activity (muscle × time), *n* is the level (or number) of muscle synergies ranging from 1 to 8, *W* is the muscle synergy structure matrix (muscle × *n*) indicating the weighting vectors of individual muscles for each muscle synergy, *C* is the muscle synergy activation matrix (*n* × time) indicating time-varying synergy activation profiles, and *e* is residual error. EMG_r_ is a reconstructed EMG matrix (muscle × time) resulting from the multiplication of *W* and *C*. The first step in our process to identify synergies underlying walking was to reconstruct EMG_o_ using NNMF with a range of 1 to 8 synergy levels (*n* = 1, *n* = 2, …, *n* = 8).

At each level, the VAF was computed as follows:

(2) $$\text{VAF}(\%)=[1-{\left({\text{EMG}}_{0}-{\text{EMG}}_{\text{r}}\right)}^{2}/{\text{EMG}}_{o}^{2}]\times 100$$

The VAF cutoff was set at 90% such that the lowest possible minimal level was the synergy number that accounted for over 90% of total VAF (Dominici et al., 2011). We defined the insufficient and excessive level as one level lower and higher than the minimal level, respectively. For example, if the minimal level was four, the insufficient level was three and the excessive level was five.

The second step was to match similar synergy structures across the 20 gait cycles at each synergy level using *k*-means clustering. Synergies were clustered using coefficients of the W matrix (i.e., an 8-dimensional feature space). In the clustering process, the value of *k* was set equal to each synergy level being analyzed.

The third step was to assess the reliability within each cluster by computing the intra-class correlation coefficient (ICC) of the synergy structure (ICCw) and activation (ICCc) matrices across the 20 sets. An inherent risk of the clustering process is the presence of large numbers of samples near the cluster boundaries, which can lead to changes in clustered groups if the analysis is run multiple times. To prevent inaccurate clustering, the combination of *k*-means clustering and ICC analysis was repeated 1000 times and the clustering result with the highest mean ICCw values across levels was selected.

Next, we combined a correlation analysis with *k*-means clustering to identify similar synergies between levels. First, *k*-means was used to cluster synergy data at each level from 1 to 8 into clusters in the 8-dimensional feature space as described above. Because *k* was set equal to each level, a single new synergy emerged at each next higher level. Next, similar synergies across levels were identified based upon proximity of each cluster centroid in the feature space. For example, to identify a new muscle synergy at level 5, each of the 20 synergy structure matrices for level 4 (8 × 4) were paired with its most frequent closest neighbor in the feature space at level 5. After matching four synergies between the level 4 and 5, the remaining unpaired synergy at level 5 was defined as the new synergy.

**FIGURE 2:**
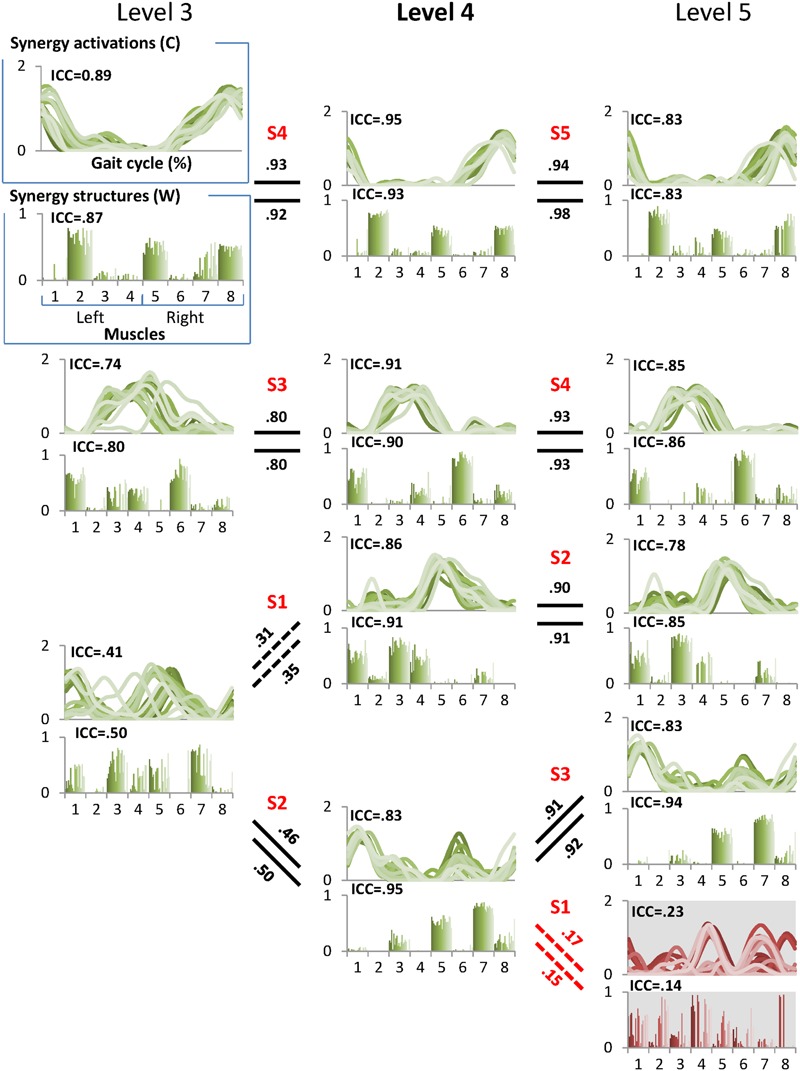
**A sample pedigree for individual muscle synergies from level 3 to 5, determined using iterative *k*-means clustering and correlation analysis.** Level 4 (bold) is the minimal level based on VAF 90% threshold. Note that this pedigree presents synergy activations (upper line graph) and synergy structures (lower bar graph) in eight muscles (1: left tibialis anterior, 2: left gastrocnemius, 3: left rectus femoris, 4: left hamstring, 5: right tibialis anterior, 6: right gastrocnemius, 7: right rectus femoris, and 8: right hamstring). Lines and bars are matched by shade, with 20 different shades representing each of 20 gait cycles. Solid lines between levels indicate similar muscle synergies between adjacent levels while dotted lines indicate a new muscle synergy as compared to lower level. Values above and beneath these lines are between level correlation (BLC) coefficients for related synergy activations (C) and structures (W), respectively. Here, synergies were assigned labels of S1-S4 or S5 based on ascending order of the correlation coefficients. The values of intra-class correlation (ICC) are shown for each synergy. The unreliable synergy determined to be noise is shown with gray background.

We then computed the between-level correlation (BLC) coefficients of synergy structure (BLCw) and activation (BLCc) matrices. The BLCw and BLCc values reported here are the averaged correlation coefficient values across the 20 EMG sets for each individual. In the case of the new synergy at the higher level, we computed BLCw and BLCc with all synergies at the lower level and matched the new synergy to the synergy at the lower level with the highest mean BLC values. In this way, we described the pedigree of muscle synergies from level 1 to 8 for each individual (**Figure 2**). Finally, we identified the correct synergy number from the pedigree as the level satisfying 90% VAF criterion and showing the highest mean ICCc and ICCw values.

Alternatively, we used only total VAF as a comparison measure for identification of correct synergy number, similar to previous studies (Clark et al., 2010; Dominici et al., 2011). For local VAF, the minimum value was selected across the eight muscles (Chvatal and Ting, 2012). Total VAF threshold was initially set at 90% as described above. Then, because total VAF threshold has varied across studies (Dominici et al., 2011; Gizzi et al., 2011; Rodriguez et al., 2013), we performed an additional analysis with VAF thresholds of 80, 85 and 95% to evaluate dependence of minimal level on this criterion. Additionally, we calculated VAF in two ways to elucidate the effect on synergy number: using concatenated EMG from the 20 consecutive gait cycles (CON) and as the average VAF across the 20 individual sets (AVE). We report the minimum and maximum VAF across the 20 gait cycles for the AVE method.

### Statistical Analysis

To evaluate the implicit assumption that gait cycles in each subject were repetitive and consistent, ICC of EMG_0_ and kinematic variables (sagittal plane hip, knee, and ankle joint excursions) was computed across the 20 gait cycles. Significance level was set at *p* < 0.05, and values are presented with means and standard deviations.

The muscle synergies within each level were ranked in ascending order based on BLCw values according to the hypothesis that noise at the excessive level (i.e., the level beyond the minimal level) would show low correlation with the reliable synergies (**Figure 2**). Thus, a synergy showing the lowest BLCw value was always assigned to the first synergy (S1). One-way repeated measures analysis of variance (ANOVA) was conducted to identify significant differences between the four indices (ICCw, ICCc, BLCw, BLCc) within the minimal, insufficient, and excessive synergy levels, respectively. One-way repeated measures ANOVA was also used to identify the differences in S1 between levels. *Post-hoc* analysis was performed by Duncan’s new multiple range test.

## Results

### Similarity of EMG and Kinematic Variables

The reliability of EMG recorded across the 20 examined strides, as measured by the ICC of EMGo, was high in most subjects (ICC = 0.86–0.95), with the exception of subject 9. The left rectus femoris of subject 9 showed low reliability (ICC = 0.39) during walking. Mean ICC values of other muscles in this subject were 0.81 ± 0.03. Reliability of the kinematic variables was of a similar range as the muscle data (0.92 ± 0.02). The reliability values for the hip, knee, and ankle joint kinematics across both legs were 0.96 ± 0.01, 0.93 ± 0.02, 0.87 ± 0.03, respectively.

### Synergy Number Based on VAF Threshold Criteria

When using the average 90% VAF cutoff criterion, four muscle synergies were required to account for bilateral muscular activations during walking for all participants except subject 5. At this threshold, minimal synergy level did not differ when extracted from 20 concatenated individual gait cycles or from an average of the 20 individual cycles (**Table 1**). The averaged total VAF values were higher (92% ± 1) than the cutoff value (90%) at level 4 in 8/9 subjects although 6/9 subjects showed that the minimal level was 3 or 5 in some cases among the 20 cycles. Since the minimal level was 4 in eight subjects, level 3 and 5 of these subjects were defined as the insufficient and excessive levels, respectively. Subject 5 required five muscle synergies (minimal level = 5) based on the VAF threshold of 90%, so level 4 and 6 were defined as the insufficient and excessive levels in this case.

**Table 1:** Total and local variance accounted for (VAF) across 20 sets at levels 3-5.

	Subject 1 (%)	Subject 2 (%)	Subject 3 (%)	Subject 4 (%)	Subject 5 (%)	Subject 6 (%)	Subject 7 (%)	Subject 8 (%)	Subject 9 (%)
Level 3								
*Total VAF*								
CON	85.0	85.9	83.3	86.1	83.9	86.3	88.4	87.9	89.3
AVE	86.2	84.9	82.1	84.2	83.0	85.3	87.5	84.7	89.8
Min.	82.0	81.7	77.2	80.6	79.1	82.0	83.3	81.1	86.2
Max.	88.9	88.1	85.7	87.8	85.4	87.2	89.4	88.8	93.8^∗^
*Local VAF*								
CON	65.4	69.7	47.7	49.3	68.5	66.3	61.8	63.7	82.4
AVE	63.1	62.8	48.1	49.8	62.0	63.7	63.9	65.0	74.5
Min.	39.5	30.3	34.9	31.9	45.7	42.1	42.2	53.1	55.7
Max.	72.6	78.2	64.4	69.1	73.4	77.6	77.2	76.9	83.1
Level 4								
*Total VAF*								
CON	**92.1**	**92.7**	**90.5**	**93.3**	89.4	**90.1**	**90.9**	**91.7**	**92.8**
AVE	**92.9**	**92.0**	**90.4**	**93.3**	89.7	**91.3**	**92.3**	**91.2**	**94.2**
Min.	90.3	89.2^†^	87.3^†^	90.5	86.9	88.5^†^	88.1^†^	88.2^†^	92.5
Max.	94.7	94.0	92.1	95.0	92.1^∗^	93.6	94.1	93.8	96.7
*Local VAF*								
CON	76.1	79.9	75.8	82.5	81.4	82.4	82.7	77.7	85.0
AVE	70.9	79.1	75.4	82.7	75.6	77.8	82.3	78.8	84.7
Min.	58.5	66.0	62.9	69.6	63.4	64.1	69.8	65.2	75.9
Max.	85.0	89.0	83.8	88.1	85.1	85.2	88.9	87.5	93.4
Level 5								
*Total VAF*								
CON	94.6	96.0	94.1	95.7	**95.3**	95.1	94.5	96.0	95.9
AVE	95.6	95.6	94.6	96.5	**94.9**	95.8	95.2	96.0	97.3
Min.	93.4	94.3	92.7	94.8	93.4	93.8	92.4	94.5	95.9
Max.	96.7	97.1	96.1	98.2	96.7	97.0	96.5	97.8	98.5
*Local VAF*								
CON	74.2	81.0	78.2	92.4	87.5	86.6	88.8	81.6	92.2
AVE	80.9	87.1	81.5	91.6	85.9	83.7	86.9	86.9	91.5
Min.	70.0	76.9	74.5	86.2	79.1	79.3	80.0	80.2	85.8
Max.	90.5	94.2	89.1	96.0	93.7	92.6	91.6	91.4	97.3

*The minimal level of individual subjects is presented with bold (cutoff: total VAF > 90%). The VAF values were determined using both consecutive (CON) and averaged (AVE) VAF. In the AVE method, minimum (Min.) and maximum (Max.) VAF values across the 20 gait cycles were also presented. Instances in which the minimal synergy level from an individual gait cycle was lower (^∗^) or higher (^†^) than the AVE and CON methods are indicated.*

**FIGURE 3:**
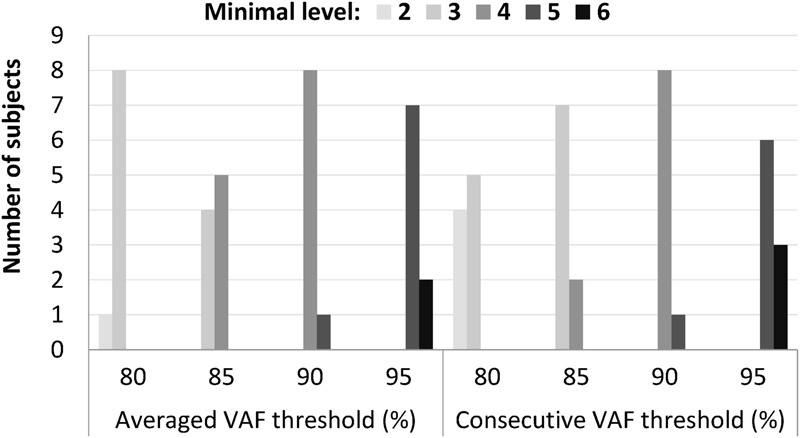
**Minimal level varied according to different cutoff values that ranged from 80 to 95%, when an absolute threshold of variance accounted for (VAF) was used to determine the number of synergies.** Among the four thresholds, 90% VAF threshold shows the most consistent minimal level between subjects in both averaged and consecutive type.

The minimal synergy level was strongly affected by the value of the VAF cutoff threshold (**Figure 3**). At VAF threshold of 80%, the minimal level was 2 or 3 for all 9 subjects for both the average and consecutive methods, while a threshold of 95% resulted in minimal level of 5 or 6 synergies.

### Identification of Reliable Synergies

**FIGURE 4:**
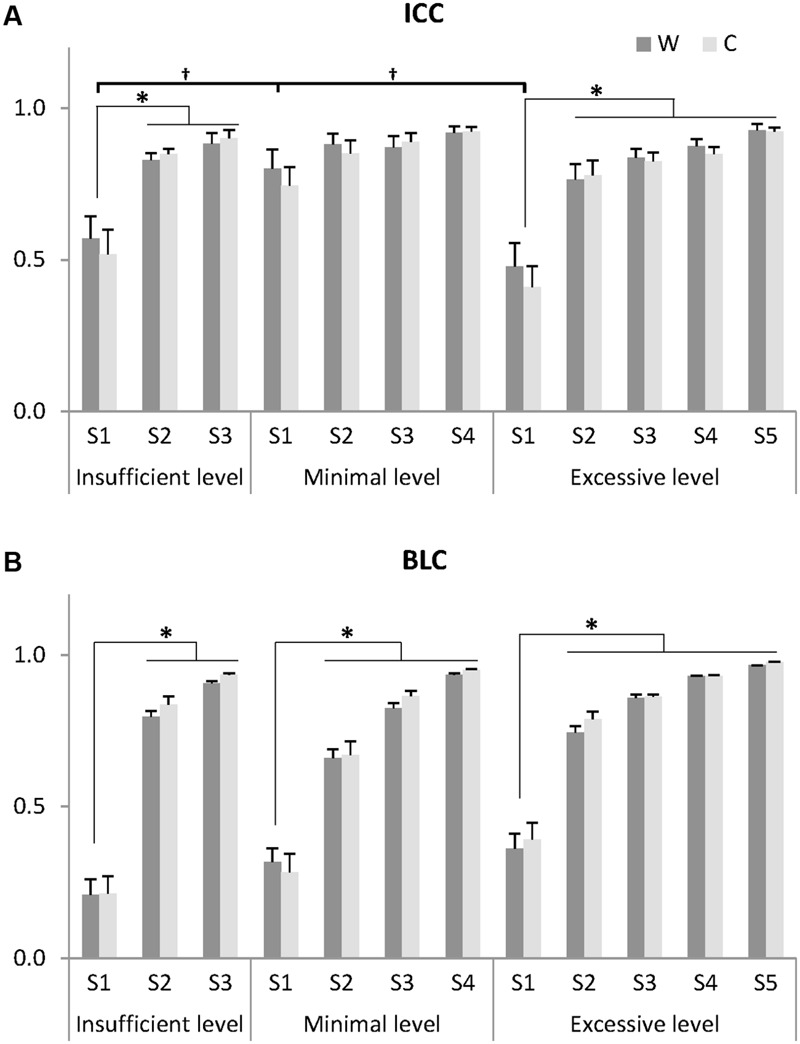
**Indices for identifying noise elements: intra-class correlation coefficient (ICC) of muscle synergies for 20 gait cycles **(A)** and between-level correlation (BLC) with lower level **(B)**.** Muscle synergies (S) at each insufficient, minimal, and excessive level were arranged by ascending order of BLC value for W. Thus, a synergy showing the lowest correlation with the lower level was assigned to S1 and a synergy showing the highest correlation was assigned to the S3, S4, and S5 at the insufficient, minimal, and excessive levels, respectively. Data presented here are the grand average across 20 gait cycles and eight subjects whose minimal level was 4. One subject (minimal level = 5) was excluded. W: muscle synergy structure matrix; C: muscle synergy activation matrix. ^∗^Significantly different from other synergies within the same level (*P* < 0.05). ^†^Significantly different from S1 at other levels (*P* < 0.05). Data are presented with mean and standard error values.

Muscle synergies across the 20 sets were clustered at each level and the reliability within the cluster was computed using ICC. Then, muscle synergies at each level were ranked based on BLCw values. The peak mean ICC value indicated that synergy level 4 contained all reliable synergies in 7/9 individuals, with the exception of subjects 5 and 6. At the minimal level as determined by VAF threshold of 90%, mean ICC values across the synergies were higher than at both insufficient and excessive levels (**Figure 4**). ICC values of synergy activation showed similar results with those of the structure.

The ICC values assigned to S1, the synergy with the lowest correlation to the previous level, showed the lowest reliability within each level in both structure and activation (**Figure 4A**). Within each level, the ICC values of S1 were significantly lower than those of other synergies in the insufficient (ICCw: *F* = 23.659, *p* = 0.001; ICCc: *F* = 12.164, *p* = 0.008) and excessive levels (ICCw: *F* = 13.108, *p* = 0.014; ICCc: *F* = 34.634, *p* = 0.002). In between-level comparisons, the ICC values of S1 at the minimal level were significantly greater than those of other levels (ICCw: *F* = 5.977, *p* = 0.013; ICCc: *F* = 6.475, *p* = 0.010). Importantly, at the minimal level, no significant differences in ICCw and ICCc were observed between S1-S4 (*p* > 0.05), suggesting that there were no unreliable components.

**FIGURE 5:**
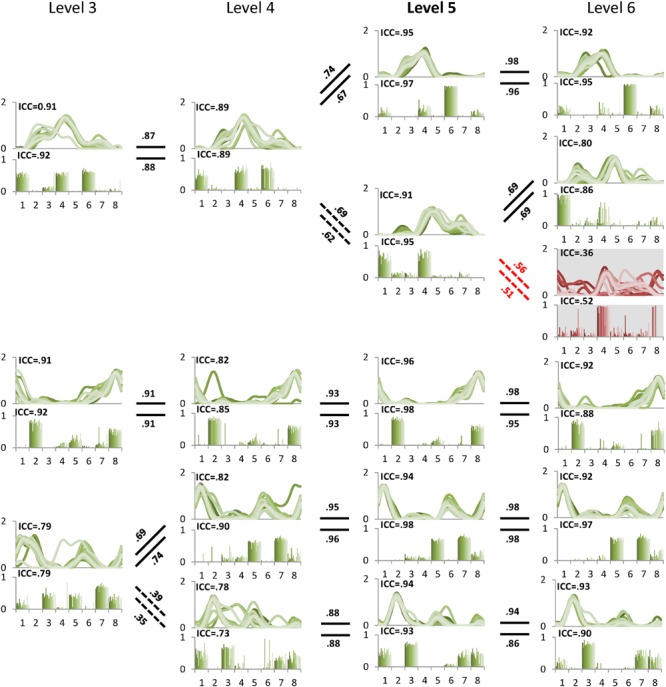
**Pedigree of subject 5.** This subject showed that level 5 (bold) was the minimal level based on VAF 90% threshold. Mean ICC values for W and C were also the highest at level 5. Unreliable element is shown at level 6 with gray background. Abbreviations and muscle numbers are the same as in **Figure 2**.

**FIGURE 6:**
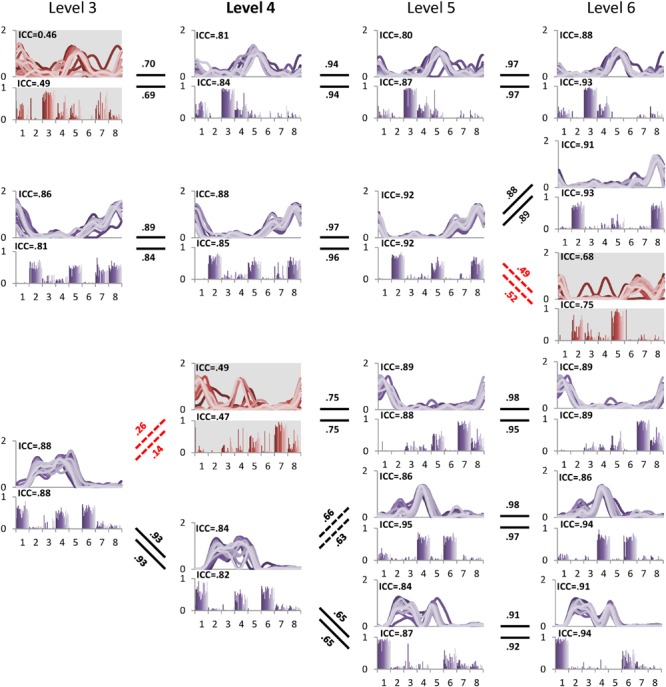
**Pedigree of subject 6, which shows a case when muscle synergies determined based on VAF threshold are not robust.** Minimal level based on VAF 90% threshold was 4 (bold level). However, one muscle synergy presented with gray background at level 4 inconsistently emerged across 20 gait cycles as shown by ICC. Muscle synergies were more reliable at level 5 with the highest ICC values compared to other levels. Abbreviations and muscle numbers are the same as in **Figure 2**.

The minimal level according to VAF threshold of 90% for subject 5 was five. Similar to level 4 for the other subjects this level had the highest ICC in synergy structure and activation (**Figure 5**). For subject 6, although the minimal level identified by 90% VAF threshold was 4, ICC values for synergy structure and activation were higher at level 5 as compared to levels 4 and 6 (**Figure 6**), indicating that level 5 is the correct minimal level and contains the most reliable muscle synergies for subject 6 although this was not the minimal level based on 90% VAF threshold. As a result of increasing one level in subject 6, total VAF increased by 5 and 4.5% for CON and AVE, respectively (**Table 1**). Subject 9 showed relatively low ICCs in both synergy structure (mean: 0.69, S1: 0.56) and activation (mean: 0.68, S1: 0.46) at the minimal level 4; however, these ICC values were higher than those at the insufficient (mean: 0.64 and 0.62, S1: 0.43 and 0.25) and the excessive (mean: 0.68 and 0.67, S1: 0.33 and 0.29) levels, so level 4 was considered correct for this individual.

The between level correlations of S1 were similar for synergy structure and activation (**Figure 4B**). Within each level, both BLCw (insufficient level: *F* = 429.314, *p* < 0.001; minimal level: *F* = 70.549, *p* < 0.001; excessive level: *F* = 269.148, *p* < 0.001) and BLCc (insufficient level: *F* = 595.246, *p* < 0.001; minimal level: *F* = 1069.360, *p* < 0.001; excessive level: *F* = 61.184, *p* = 0.001) were significantly less than the other synergies. In contrast, neither BLCw (*p* > 0.05) nor BLCc (*p* > 0.05) of S1 were significantly different between levels. By describing the pedigree for each level (**Figure 2**), our data driven, *k*-means clustering and subsequent correlation analysis consistently found a pattern whereby when progressing to a higher level, two synergies would bifurcate from one synergy at the lower level while the other synergies were preserved with high between-level correlation. This pattern was identified while progressing between all levels.

## Discussion

In this work, we present iterative *k-*means clustering with ICC and BLC as a quantitative method for more precise identification of synergy number during walking. The level containing reliable synergies was identified from the peak ICC values as a function of increasing synergy number. In all cases, one synergy in the excessive level showed poor stride-to-stride reliability (low ICC); thus an unreliable synergy that was rarely recreated across gait cycles was present when synergy number was overestimated. This unreliable synergy also showed the lowest BLC indicating low correlation with all reliable synergies. We consider this synergy to be a noise element conceptualized in previous studies (Cheung et al., 2005; Chvatal and Ting, 2012).

Our analysis confirmed that synergy number is highly dependent upon the selected VAF threshold (**Figure 3**). Our results show that a more nuanced approach than application of a constant VAF cutoff threshold across a group of subjects is required to precisely extract synergies underpinning walking, and we propose ICC and BLC as more accurate discriminators of reliable and unreliable synergies. For example, subject 6 showed that ICC values peaked at level 5 and were reduced at level 6. We conclude that this subject has five synergies despite level 4 satisfying the 90% VAF criterion. We note that for most subjects, ICC values were highest at the minimal level determined by 90% VAF threshold, but this would not have been the case if the a VAF threshold of 80 or 95% was selected. To our knowledge, there is no evidence why the same VAF criterion should be applied for all individuals.

Other studies have utilized muscle synergy analysis without applying a criterion to determine synergy number required to describe a movement. For example, Steele et al. developed the dynamic motor control index during walking (walk-DMC) that quantifies the unexplained variance beyond the first synergy level (Steele et al., 2015). While this index has been shown to correlate with gross measures of function and may therefore be useful as a clinical measure, it does not capture the full motor control strategy utilized during walking. Similarly, Clark et al. (2010) examined motor complexity in individuals who had strokes by extracting synergies at level 4 regardless of VAF. Their analysis revealed that activation timing between modules overlapped at this level in those post-stroke, indicating a reduced level of complexity. Similar to the walk-DMC, such an approach could be useful clinically to quantify locomotor complexity, but it does not enable examination of individual motor control strategies for walking with the same resolution as approaches which identify all reliable motor modules.

Incorporation of clustering and ICC using single stride analysis enables exploration of trial-to-trial variability of synergies. For example, subject 9 presented lower ICC than other subjects, indicating higher variability in the extracted muscle synergies. Closer examination attributed the low ICC to poor repeatability of left rectus femoris activations, a result that was not due to measurement noise but instead reflected stride-to-stride variability in muscle activation. Although unique in our study cohort, previous reports have described high stride-to-stride variability of rectus femoris in normal walking (Winter and Yack, 1987; Annaswamy et al., 1999). Either way, high variability of muscle synergies warrants further exploration, especially when applying synergy analyses to individuals with brain injury, because such variability across repetitions of the same movement could be interpreted as a challenge to the synergy hypothesis which postulates that synergies represent a fixed modular pattern of motor activities in purposeful behavior (Ivanenko et al., 2006; Bizzi and Cheung, 2013). Increased motor variability across trials is often observed in patients with brain injuries (Kim et al., 2016) or older adults (Lovden et al., 2007). Moreover, muscle activity patterns can vary owing to changes in sensory input and subcortical reflexes (Sinkjaer et al., 2000; Kim et al., 2013). The method introduced here does not place a threshold on synergy consistency (ICC) but instead utilized a local peak in ICC in combination with a VAF threshold to identify the most reliable minimal level. While we see no reason why this method would not be similarly effective regardless of the population, further studies are necessary to validate this approach in clinical populations, such as those with stroke or cerebral palsy, different age groups, or healthy individuals performing novel tasks that may show more variability than the cohort in this study.

Our method was designed as a tool to enhance precision when extracting the fundamental synergies that describe muscle activity during walking. Bootstrapping methods which randomly sample individual strides may account for variability when determining synergy number or minimal level (Cheung et al., 2009; Sawers et al., 2015), yet the cause of this variability may not be clear. The ICC value introduced quantifies consistency of individual muscle synergies independent of the VAF value or bootstrapping confidence interval. In this way, our approach can aid in determining whether an elevated minimal level is due to reduced consistency of a subset of deployed synergies or to a more complex motor control strategy. Given that the number of extracted synergies required to describe walking has been correlated clinically with functional capability (Bowden et al., 2010), precision is paramount and our method would be beneficial for such analyses.

However, our method may not identify all possible synergies which could be deployed during walking. For example, synergies that arise from or are distorted by a reflexive response or those which only contribute occasionally to the movement may not be identified due to high stride-to-stride variability. Similarly, inconsistent synergies that arise during development or early learning of a skill, or that result from neurological impairments may result in reduced ICC values. However, as discussed above, in our approach there is no set threshold on ICC required to define the minimal synergy level. We also note that our *k*-means clustering and correlation analysis does not, a priori, assume the existence of synergies. Indeed, it is possible that poor reliability (low ICC/BLC) would be present at all levels, in which case the minimal level would be equal to the number of muscles which would suggest that either there were no reliable synergies or that task performance across repetitions (e.g. strides) was not consistent.

In our sample of healthy adults, muscle activity during walking was accounted for with four or five synergies in agreement with previous studies of bilateral muscle activity (Dominici et al., 2011; Maclellan et al., 2014). The number of synergies may reflect the size of one’s motor repertoire or motor complexity (Ting et al., 2015) so variation across individuals is not surprising. Even in an everyday task such as walking, there may be differences in performance levels across individuals. Evidence that expert training such as ballet increases the number of muscle synergies shared across different walking tasks (Sawers et al., 2015) further supports this interpretation.

The muscle synergies in this study were extracted from EMG recorded bilaterally during walking. Although extraction of synergies from unilateral EMG during walking is common, our results align with previous studies that have extracted bilateral muscle patterns in children and adults (Dominici et al., 2011). It has been shown that bilateral synergies reveal more consistent synergies between strides as compared to unilateral sets (Maclellan et al., 2014). While these studies, as well as computational models of central pattern generators in animals (Sherwood et al., 2011), support the role of bilateral coordination during walking, future studies will also examine the performance of our synergy extraction techniques in larger muscle sets and will compare its performance in unilateral versus bilateral EMG datasets.

Our data driven approach consistently found that unreliable synergies had weak correlations with the synergies of the lower level. That is, an unreliable synergy, denoted as S1 in this paper, always shows the lowest BLC value. Thus, the BLC is a useful metric for identifying an unreliable synergy at the excessive level. The synergy pedigree identified using clustering and between-level correlation analysis presents a bifurcation pattern observed in all subjects whereby one synergy at the lower level splits into two synergies at the next level. Although further studies with additional muscles are necessary to confirm this pattern, the same phenomenon has been identified in previous studies in animals and humans (D’Avella et al., 2003; Danner et al., 2015). This pattern supports the concept of synergy merging when moving from a higher to lower level. Yet, because the emergent synergy (S1) constituted an unreliable (or noise) element at the excessive level (**Figure 2**), functional interpretations of merging or splitting of synergies should be carefully examined. It has been postulated that the reduced synergy number in individuals with brain injury arises from merging of several synergies, a result that may indicate reduced complexity of neural control signals (Clark et al., 2010; Allen et al., 2013; Rodriguez et al., 2013). While this may indeed be the case, our results demonstrate the possibility that merging is also caused by inaccurate or underestimation of the minimal level. In all subjects here, one synergy at the insufficient level was always formed from merging of two at the minimal level, suggesting examination of synergies beyond VAF threshold is necessary to confirm the reduction from unimpaired individuals.

An increase in synergy number and refinement of their structure during infant motor development has been hypothesized by comparing neonatal and adult groups (Dominici et al., 2011). Neonates demonstrated two muscle synergies while adults showed four, leading to the theory that the number of muscle synergies are related to maturity of the central nervous system. The presence of fewer synergies in those with motor disabilities is also not surprising (Clark et al., 2010) because they have diminished coordination compared to non-injured adults. However, neonates and those with movement disorders are likely to have characteristics that affect VAF, such as increased motor variability, muscle weakness, and reduced modulation of muscle activity. Our proposed methods for identifying reliable synergies may provide a useful tool for enhancing precision of synergy analysis in these populations.

One potential limitation of this study is the use of eight bilateral EMG channels; however, a previous study indicated that the weight (W) and activation (C) matrices of synergies derived from factorization algorithms are highly similar when using a range of 6 to 192 EMG channels (Muceli et al., 2014), suggesting that the current setup is useful for demonstration of our algorithm. Experiments are planned in the future to confirm its use with a larger muscle set.

In summary, the central nervous system may modularly recruit groups of muscles via a single command, termed a muscle synergy, to execute coordinated movements. Recent evidence suggests individuals with neurological deficits may possess fewer synergies, indicating reduced motor complexity. Yet, many studies rely on a basic metric, percentage of VAF by the extracted synergies, to quantify the number involved in a motor task. We confirmed that a single threshold applied across individuals can lead to spurious results. In addition to the VAF threshold criteria, we introduce novel measures to enhance precision when distinguishing reliable synergies from unreliable elements during walking. When applying the 90% VAF criterion, we found peak ICC values at level four or five, depending on the individual. Within each individual, the next synergy level beyond the one with peak ICC (i.e., the excessive level) retained high VAF values, yet unreliable (noise) synergies emerged as indicated by reduced ICC values compared to the minimal level. Our analysis demonstrates that reliable and unreliable synergies emerge in the same way, and thus careful analysis is required to examine motor control complexity especially in the population having high motor variability.

## Author Contributions

Conception and design of the experiments: YK, TB. Collection, analysis and interpretation of data: YK, TB, DD. Drafting and revising the article: YK, TB, DD. Final approval of the version to be published: YK, TB, DD.

## Conflict of Interest Statement

The authors declare that the research was conducted in the absence of any commercial or financial relationships that could be construed as a potential conflict of interest.
